# Supplementary material for: MHC Class II Expression Influences the Composition and Distribution of Immune Cells in the Metastatic Colorectal Cancer Microenvironment
Source: Cancers (Basel). 2022 Aug 24;14(17):4092. doi: 10.3390/cancers14174092 (PMC9454847; doi:10.3390/cancers14174092)

Supplemental Table S1: *Antibody Data.* Antibodies used in multiplexed staining including primary, dilution, secondary antibody, catal OPAL TSA™ fluorophore.

Primary Antibody	Company and Catalogue Number	Dilution	Secondary Antibody	Catalogue Number
**CD3**	Dako<br>A0452	1:400	Opal Polymer®	ARH1A01EA
**CD8**	SpringBio<br>M5390	1:400	Opal Polymer®	ARH1A01EA
**FoxP3**	CST<br>12653	1:400	Opal Polymer®	ARH1A01EA
**CD163**	Leica<br>Lot 6080491	1:400	Opal Polymer®	ARH1A01EA
**PD-L1**	CST<br>13684	1:200	Opal Polymer®	ARH1A01EA
**Panycytokeratin**	Dako<br>M3515	1:500	Opal Polymer®	ARH1A01EA
**HLA-DR**	Santa Cruz<br>SC-53319	1:4000	Opal Polymer®	ARH1A01EA
**Ki-67**	Abcam<br>Ab15580	1:600	Opal Polymer®	ARH1A01EA
**Granzyme B**	Abcam<br>Ab4059	1:100	Opal Polymer®	ARH1A01EA

Supplemental Figure S1 - *Association of HLA-DR expression on survival, MSI status, and interactions among regulatory and cytotoxic T cells*

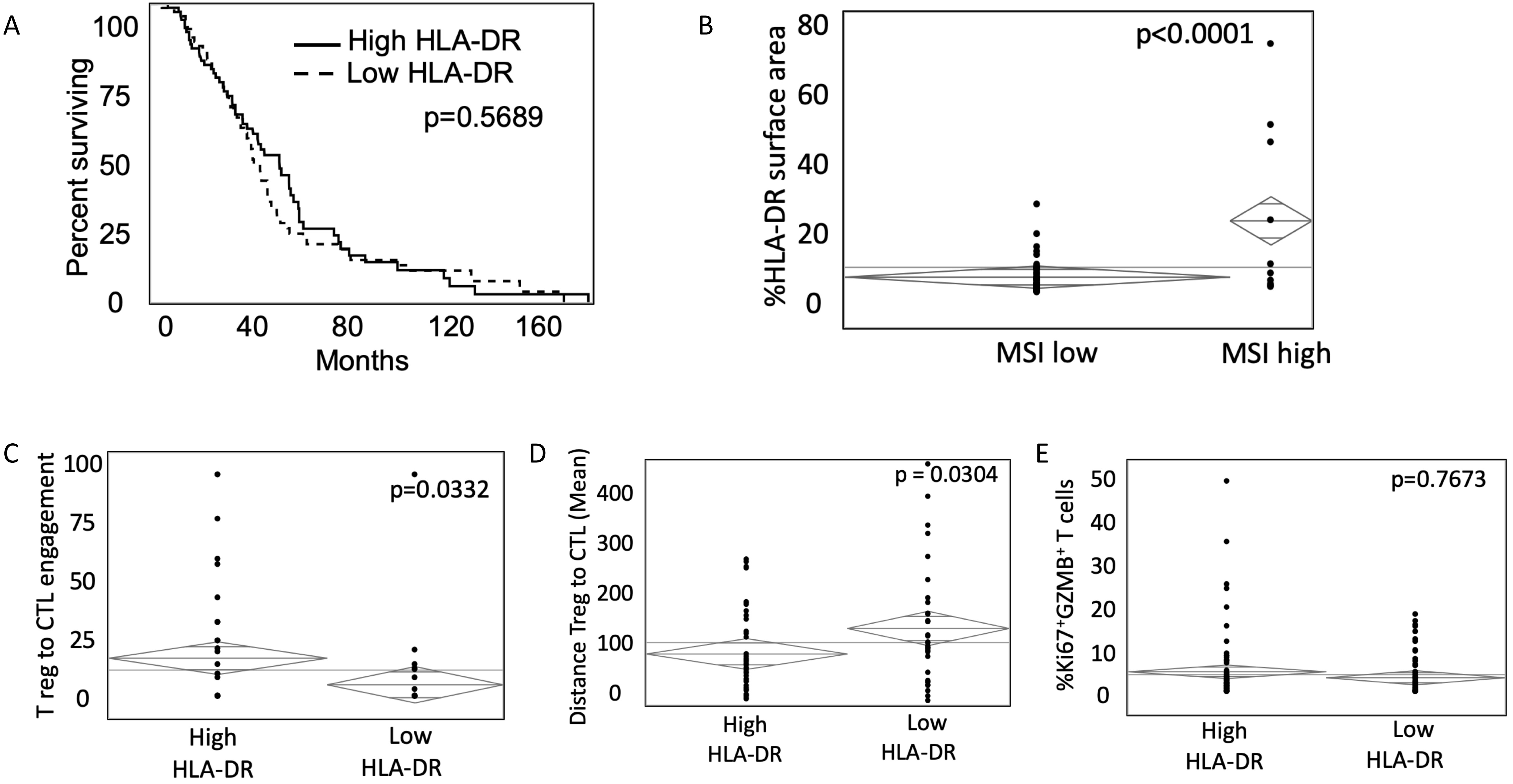

Supplement: Supplementary file 1 [file cancers-14-04092-s001.zip › cancers-1831077-supplementary.pdf]
